# Prevalence and factors associated to chronic kidney disease in older adults

**DOI:** 10.11606/S1518-8787.2019053000727

**Published:** 2019-04-26

**Authors:** Thatiana Lameira Maciel Amaral, Cledir de Araújo Amaral, Maurício Teixeira Leite de Vasconcellos, Gina Torres Rego Monteiro

**Affiliations:** I Universidade Federal do Acre . Centro de Ciências da Saúde e do Desporto . Programa de Pós-Graduação em Saúde Coletiva . Rio Branco , AC , Brasil; II Instituto Federal de Educação, Ciência e Tecnologia do Acre . Campus Rio Branco. Rio Branco , AC , Brasil; III Fundação Instituto Brasileiro de Geografia e Estatística . Escola Nacional de Ciências Estatísticas . Rio de Janeiro , RJ , Brasil; IV Fundação Oswaldo Cruz . Escola Nacional de Saúde Pública Sérgio Arouca . Rio de Janeiro , RJ , Brasil

**Keywords:** Aged, Renal Insufficiency, Chronic, epidemiology, Risk Factors, Comorbidity, Health Surveys, Idoso, Insuficiência Renal Crônica, epidemiologia, Fatores de Risco, Comorbidade, Inquéritos Epidemiológicos

## Abstract

**OBJECTIVE:**

To verify the prevalence of chronic kidney disease and the factors associated to it in older adults (≥ 60 years).

**METHODS:**

This is a population-based research conducted in 2014, involving 1,016 older adults living in urban and rural areas of the municipality of Rio Branco, Acre. Chronic kidney disease was defined by glomerular filtration rate < 60 ml/min/1.73 m ^2^ , estimated by the equations of the Chronic Kidney Disease Epidemiology Collaboration, and the presence of albuminuria > 29 mg/g. Association measure were estimated by gross and adjusted odds ratio (OR), with a confidence level of 95% (95%CI).

**RESULTS:**

The overall prevalence of chronic kidney disease was 21.4% in older adults, with the associated factors age, diabetes (OR = 3.39; 95%CI 2.13–5.40), metabolic syndrome (OR = 2.49; 95%CI 1.71–3.63), self-assessment of poor health (OR = 1.79; 95%CI 1.10–2.91), arterial hypertension (OR = 1.82; 95%CI 1.04–3.19) and obesity (OR = 1.69; 95%CI 1.02–2.80).

**CONCLUSIONS:**

The prevalence of chronic kidney disease was high in older adults, being associated with age, self-assessment of health as bad or very bad, obesity, diabetes and metabolic syndrome.

## RESUMO

**OBJETIVO:**

Verificar a prevalência de doença renal crônica e os fatores a ela associados em idosos (≥ 60 anos).

**MÉTODOS:**

Trata-se de um inquérito de base populacional realizado em 2014, envolvendo 1.016 idosos residentes nas zonas urbana e rural do município de Rio Branco, Acre. A doença renal crônica foi definida pela taxa de filtração glomerular < 60 ml/min/1,73 m ^2^ , estimada pelas equações da *Chronic Kidney Disease Epidemiology Collaboration* , e presença de albuminúria > 29 mg/g. Medidas de associação foram estimadas por regressão logística (OR) bruta e ajustada, com grau de confiança de 95% (IC95%).

**RESULTADOS:**

A prevalência geral de doença renal crônica foi de 21,4% nos idosos, com os fatores associados idade, diabetes (OR = 3,39; IC95% 2,13–5,40), síndrome metabólica (OR = 2,49; IC95% 1,71–3,63), autoavaliação de saúde ruim (OR = 1,79; IC95% 1,10–2,91), hipertensão arterial (OR = 1,82; IC95% 1,04–3,19) e obesidade (OR = 1,69; IC95% 1,02–2,80).

**CONCLUSÕES:**

A prevalência de doença renal crônica foi alta entre os idosos, estando associada com idade, autoavaliação de saúde como ruim ou muito ruim, obesidade, diabetes e síndrome metabólica.

## INTRODUCTION

The pattern of morbimortality due to chronic kidney disease (CKD) in underdeveloped and developing countries is changing due to the transition of infectious diseases to chronic non-communicable diseases ^1^ . Prevalence projections of CKD in the U.S. for 2020 and 2030, between individuals over 30 years, estimate that the disease will go from 13.2% in 2010 to 14.4% in 2020 and to 16.7% in 2030 ^2^ .

The prevalence of CKD changes with age, reaching higher values among older people, and may vary from 25.1% in Nicaragua, in the age group of 60 to 70 years ^3^ , to 30.8% in Canada, among those aged ≥ 65 years ^4^ . The reduction in the glomerular filtration rate (GFR) for less than 60 ml/min/1.73 m ^2^ might be attributed to aging, which causes progressive structural and functional changes in the kidneys, or as consequence of the presence of comorbidities and of the exposition of risk factors throughout life ^5^ .

Among older adults, the presence of CKD represents an increased risk for multiple events adverse to health that may culminate in death, and the early detection of the reduced GFR and albuminuria is important to assist in the therapeutic decision-making and consequent reduction of complications ^6^ . Thus, this study aimed to estimate the prevalence of CKD and the factors associated to it in older adults (60 years or more), using part of the data from the *Estudo das Doenças Crônicas* (Edoc – Study of Chronic Diseases).

## METHODS

The Edoc is composed of two household surveys: the Edoc-A, on adults (18 to 59 years-old), and the Edoc-I, on older adults (60 years or more), all living in Rio Branco, Acre. Individuals with cognitive impairments that could preclude communication or understanding of the questions were excluded. Sampling plans were selected in two stages, census enumerations areas (CEAs) and households, being the first common to the two researches. The CEAs were selected with probability proportional to the number of private households in the 2010 Demographic Census (CD2010) of the Brazilian Institute of Geography and Statistics (IBGE). The households were selected by systematic sampling with random starts and distinct ranges per survey. In the households selected for Edoc-I, all the older adult residents were interviewed.

The sample size was estimated considering the prevalence of renal function changes of 40% in older adults ^7^ , with confidence level of 95% and absolute error of 3% for the simple random sampling of proportions. Considering that the sampling plan is conglomerated by CEA, a sampling plan effect of 1.95 was arbitrated to determine the sample size, which received an increase of 12.5% to compensate the expected non-responses. This procedure resulted in a sample of 1,148 older adults. By dividing this sample size by the number of older adults per household, according to CD2010, and by defining the selection of 73 households per CEA, a sample size of CEAs of 40 was obtained. The effective sample was 1,016 older adults.

The sampling weights were calculated by the inverse of the inclusion probabilities in each stage and were subsequently calibrated for populational data by sex and age groups, using a post-stratification estimator, in order to address these typical biases of the household surveys and to correct differential non-responses ^8^ . The population data used in the calibration of the sampling weights were estimated for July 1, 2014, using the linear trend method ^9^ that the IBGE applies in its population estimations per municipality. This study included all who performed laboratory assessments of serum creatinine, that is, 983 older adults (578 women and 405 men), that correspond to a subsample of complete information on the topic. For more details about the sampling plan of the Edoc, estimation and calibration of sample weights and subsamples, see the article by Amaral et al. ^10^ .

A total of 33 participants were excluded from the analysis of renal function due to the lack of information on serum creatinine. Each household with a participant resident filled a form containing general information on housing, sanitation and family. Individually, a structured form was used in thematic modules with socioeconomic, demographic, behavioral and health information.

In the physical assessment, the anthropometric data included the measurement of weight, height and circumferences of the waist, hips, arm and calf, all in duplicate, and the average of the measurements was considered.

Blood pressure (BP) was measured according to the protocol recommended by the Brazilian Society of Cardiology, which recommends measuring 30 minutes or more after the last drank dose of caffeine or smoked cigarette, in triplicate: one after five minutes of initial rest and two others in intervals of two minutes ^11^ .

The biological material was analyzed in the same laboratory to ensure the standardization of methods. Blood samples were obtained by peripheral blood collection, with prior antisepsis of the antecubital fossa of the participants. The serum extracted was stored for biochemical dosage of triglycerides, total cholesterol and fractions: high-density lipoprotein (HDL) and low-density lipoprotein (LDL). Serum creatinine was dosed by traceable enzymatic method of isotope dilution mass spectrometry (IDMS) in an automatic analyzer (Labmax 240 Premium). To analyze serum glycemia, a 4 ml blood sample was used, stored in a vacuum tube containing 2 mg/ml of sodium fluoride centrifuged before analysis. The serum glycemia was dosed by the glucose oxidase method (Labtest Diagnostica).

For the urine sample, 50 ml of the midstream of first morning urine of each individual were collected, subsequently processed for physicochemical and microscopic analysis of the sediment.

Urinary creatinine was dosed by the enzymatic Trinder method (Kit Creatinina Enzimática, Labtest), and albuminuria by the immunoturbidimetric method (Kit Turbiquest Plus, Labtest). Albuminuria is defined with a ratio of albumin to creatinine of 30 mg/g or more, being the value of 30 to 299 considered A2, or moderately increased, and the value ≥ 300 mg/g considered A3, or markedly increased.

The systemic arterial hypertension (SAH) was defined as diastolic blood pressure (DBP) ≥ 90 mmHg, systolic blood pressure (SBP) ≥ 140 mmHg and/or current use of anti-hypertensive medication ^11^ .

The presence of diabetes was defined according to the criteria of the American Diabetes Association (ADA): fasting plasma glucose ≥ 126 mg/dL or use of oral hypoglycemic or insulin ^12^ .

Dyslipidemia was defined by abnormal levels of one or more of the following lipid blood components: triglycerides ≥ 150 mg/dL, total cholesterol ≥ 200 mg/dL, LDL ≥ 160 mg/dL, HDL in men < 40 mg/dL and in women < 50 mg/dL, in addition to the history of use of medications to reduce these values. For individuals aged less than 20 years, the cut-off points are: triglycerides ≥ 130 mg/dL, total cholesterol ≥ 170 mg/dL, LDL ≥ 130 mg/dL and HDL ≤ 45 mg/dL ^13^ .

For the diagnosis of metabolic syndrome (MS), the I Brazilian Guideline of Diagnosis and Treatment of Metabolic Syndrome was used, which requires the presence of at least three of the following elements: waist diameter > 102 cm for men and > 88 cm for women; triglycerides ≥ 150 mg/dL; HDL < 40 mg/dL for men and < 50 mg/dL for women; SBP ≥ 130 mmHg, DBP ≥ 85 mmHg or use of anti-hypertensive; fasting glycemia ≥ 110 mg/dL or use of hypoglycemic ^14^ .

The dependent variable of the study (CKD) was defined according to the formulas of the Chronic Kidney Disease Epidemiology Collaboration (CKD-EPI), when GFR < 60 ml/min/1.73 m^2^ and/or albuminuria > 29 mg/g ^15^ .

The use of equations to estimate the GFR provides adjustments for the substantial variations regarding sex, age, body surface and ethnicity, characteristics that interfere in the production of creatinine. To this end, the validated equations of the Chronic Kidney Disease Epidemiology Collaboration (CKD-EPI) were used, described below:

Female:

Black skin color ≤ 0.7 = 166 × (serum creatinine/0.7) ^-0.329^ × 0.993 ^age^

Black skin color > 0.7 = 166 × (serum creatinine/0.7) ^-1.209^ × 0.993 ^age^

White skin color ≤ 0.7 = 144 × (serum creatinine/0.7) ^-0.329^ × 0.993 ^age^

White skin color > 0.7 = 144 × (serum creatinine/0.7) ^-1.209^ × 0.993 ^age^

Male:

Black skin color ≤ 0.9 = 163 × (serum creatinine/0.9) ^-0.411^ × 0.993 ^age^

Black skin color > 0.9 = 163 × (serum creatinine/0.9) ^-1.209^ × 0.993 ^age^

White skin color ≤ 0.9 = 141 × (serum creatinine/0.9) ^-0.411^ × 0.993 ^age^

White skin color > 0.9 = 141 × (serum creatinine/0.9) ^-1.209^ × 0.993 ^age^

The category “black skin color” included the individuals who self-declared black or brown.

In 2012, the categories of risk were defined for the progression of chronic kidney disease based on serum creatinine, with correction by formulas for obtaining GFR, and in albuminuria. In addition, the stage 3 of the CKD was divided in 3a and 3b. An important emphasis of the guidelines is the adoption of categories of CKD prognosis, classifying from low to high risk of progression of the acute renal injury for CKD and other complications ^15^ .

The data was analyzed using the Complex samples routines of the Statistical Package for the Social Sciences software (SPSS), version 20.0, for Windows. The natural weight of the design, the sample selection stratum, the primary sampling unit code (CEA) and the calibrated weight were maintained in the data files.

The data were analyzed in a descriptive and exploratory way to assess the distribution and characterize the studied population. The qualitative variables were described in absolute numbers and proportions. To analyze the differences between categorical variables, we used Pearson’s chi-squared test.

A bivariate analysis was also performed in order to explore the association of the different variables with the object of study. Models of odds ratio estimated the magnitude of association between the dependent variable CKD and the independent variables. For multiple analysis, the variables included were the ones that showed a p value lower than 0.10 in the crude analysis, and the magnitude of the variables adjusted by the other significant variables was analyzed. The backward method was used in the selection of the variables in the multivariate analysis. Age and sex interactions with CKD were tested, with no effect changes observed. The significance level considered was α = 0.05.

All analysis considered the effect of the sampling design and the calibrated weights of the observations, and the results of the observations are demonstrated by “n” and the results considering the calibrated weights for extrapolation for the population by “expanded n (N)”. To this end, the pseudo maximum likelihood method (PML) was used, considering the sampling weights and the structural information of the sampling plan. The interferences were assessed by Wald statistics based on the sampling plan, along with the F distribution.

This study meets the requirements of Resolution CNS 466/2012, which addresses ethics in research involving human beings, and was approved by the Research Ethics Committee of the Universidade Federal do Acre, under CAAE: 17543013.0.0000.5010.

## RESULTS

The prevalence of CKD was of 21.4%, with verified decreased GFR in 13.0% of older adults and the presence of albuminuria in 11.6%. The prevalence of the prognosis with mild, moderate and high risk was, respectively, 14.5%, 4.9% and 2.1% (Table 1).

**Table 1:** Prevalence by category of risk of the prognosis of CKD evolution assessed by GFR (estimated by the CKD-EPI formula) and albuminuria in older adults from Rio Branco, state of Acre, 2014.

Categories of risk of CKD		Total		Albuminuria						CKD (GFR* and albuminuria)	

				A1 (< 30)		A2 (30–299)		A3 (≥ 300)			
				
		n	N (%)	n	N (%)	n	Exp N (%)	n	N (%)	n	N (%)
1	≥ 90	296	7,982 (34.0)	270 ^a^	7,252 (31.2) ^a^	19 ^b^	518 (2.2) ^b^	4 ^c^	116 (0.5) ^c^	23	634 (2.7)
2	60–89	547	12,407 (53.0)	486 ^a^	10,982 (47.3) ^a^	45 ^b^	1,059 (4.6) ^b^	11 ^c^	260 (1.1) ^c^	56	1,319 (5.7)
3a	45–59	106	2,271 (9.7)	85 ^b^	1,778 (7.7)	15 ^c^	358 (1.6) ^c^	6 ^d^	135 (0.6) ^d^	106	2,217 (9.7)
3b	30–44	26	552 (2.4)	19 ^c^	385 (1.7) ^c^	4 ^d^	93 (0.4) ^d^	3 ^d^	73 (0.3) ^d^	26	552 (2.4)
4	15–29	4	93 (0.4)	4 ^d^	93 (0.4) ^d^	-	-	-	-	4	93 (0.4)
5	< 15	4	111 (0.5)	1 ^d^	24 (0.1) ^d^	2 ^d^	54 (0.2) ^d^	1 ^d^	33 (0.1) ^d^	4	111 (0.5)
Total		983	23,416 (100.0)	865	20,514 (88.4)	85	2,082 (9.0)	25	617 (2.6)	219	4,979 (21.4)

N: expanded n from sampling weights and design; %: proportion from N; CKD: chronic kidney disease; GFR: glomerular filtration rate

* CKD-EPI formula: Chronic Kidney Disease Epidemiology Collaboration (ml/min/1.73 m ^2^ ).

^a^ Low.

^b^ Mild.

^c^ Moderate.

^d^ High.

The presence of CKD was higher among octogenarian individuals, with a prevalence of almost 40.0%, and among those declared widows/widowers, who showed a prevalence of 25.9%, with statistically significant differences (Table 2).

**Table 2:** Prevalence of CKD according to sociodemographic characteristics and life habits of older adults in Rio Branco, state of Acre, 2014.

Variable	Total		CKD						p

			Yes			No			
		
	n	N	n	N	%	n	N	%	
Sex									0.519
Female	577	12,496	123	2,570	20.6	454	9,926	79.4	
Male	406	10,920	96	2,409	22.1	310	8,511	77.9	
Age group (years)									< 0.001
60–69	475	13,394	75	2,134	15.9	400	11,260	84.1	
70–79	339	6,687	80	1,586	23.7	259	5,101	76.3	
80 and more	169	3,335	64	1,259	37.8	105	2,076	62.2	
Skin color									0.158
White	241	5,601	62	1,374	24.5	179	4,227	75.5	
Brown	634	15,221	140	3,217	21.1	494	12,004	78.9	
Other	108	2,594	17	388	15.0	91	2,206	85.0	
Education level*									0.804
Higher education	38	954	10	231	24.2	28	723	75.8	
High school	112	2,828	21	528	18.7	91	2,300	81.3	
Elementary school	360	8,721	76	1,807	20.7	284	6,914	79.3	
Illiterate/Literate	465	10,712	109	2,344	21.9	356	8,368	78.1	
Marital status*									0.011
Married	365	9,144	80	1,994	21.8	285	7,150	78.2	
Single	178	4,356	28	591	13.6	150	3,765	86.4	
Widow/Widower	317	6,793	85	1,754	25.8	232	5,039	74.2	
Separated/Divorced	117	2,980	25	616	20.7	92	2,365	79.3	
Physical activity									0.153
Yes	149	3,654	26	593	16.2	123	3,061	83.8	
No	834	19,762	193	4,386	22.2	641	15,376	77.8	
Smoking									0.148
Non-smoker	282	6,696	71	1,618	24.2	211	5,078	75.8	
Ex-smoker	537	12,541	119	2,689	21.4	418	9,852	78.6	
Smoker	164	4,179	29	672	16.1	135	3,507	83.9	
Consumption of alcoholic beverage									0.149
No	857	20,138	185	4,131	20.5	672	16,007	79.5	
Yes	80	2,175	23	600	27.6	57	1,575	72.4	

N: expanded n from sampling weights and design; %: proportion from N; χ ^2^ = p: Pearson’s chi-squared test; CKD: chronic kidney disease

* The differences in relation to the total are due to the lack of information in the variable.

In the analysis of the health conditions of older adults with CKD, 29.5% reported considering their health bad or very bad, 26.4% were classified as obese, 24.0% had SAH and 22.7% had dyslipidemia. Diabetes obtained the highest prevalence, 41.5%, and metabolic syndrome was present in 30.3% of the people classified as having a CKD. The use of medications was reported by 22.6% of these older adults (Table 3).

**Table 3:** Prevalence of CKD according to health conditions in older adults in Rio Branco, state of Acre, 2014.

Variable	Total		CKD						p

			Yes			No			
		
	n	N	n	N	%	n	N	%	
Self-assessment of health									0.014
Very good/Regular	824	19,774	170	3,906	19.8	654	15,868	80.2	
Bad/Very bad	159	3,642	49	1,073	29.5	110	2,569	70.5	
BMI (kg/m ^2^ )*									0.031
< 25.0	333	7,844	59	1,275	16.3	274	6,569	83.7	
25.0–29.9	367	8,816	83	1,944	22.1	284	6,872	77.9	
≥ 30.0	248	5,932	69	1,565	26.4	179	4,367	73.6	
Hypertension*									0.008
No	231	5.571	32	728	13.1	199	4,843	86.9	
Yes	744	17,618	186	4,231	24.0	558	13,387	76.0	
Diabetes*									< 0.001
No	820	19,501	152	3,381	17.3	668	16,120	82.7	
Yes	161	3,848	67	1,599	41.5	94	2,249	58.5	
Dyslipidemia*									0.032
No	198	4,753	32	743	15.6	166	4,010	84.4	
Yes	784	18,629	187	4,236	22.7	597	14,393	77.3	
Metabolic syndrome*									< 0.001
No	618	14,842	105	2,385	16.1	513	12,458	83.9	
Yes	356	8,363	111	2,536	30.3	245	5,827	69.7	
Other self-reported CVD (CHF, MI, CVA, arrhythmia)*									0.462
No	830	19,834	182	4,132	20.8	648	15,702	79.2	
Yes	138	3,219	34	766	23.8	104	2,453	76.2	
Use of medicines									0.034
No	231	5,934	42	1,020	17.2	189	4,914	82.8	
Yes	752	17,482	177	3,960	22.6	575	13,522	77.4	
Hospitalization in the past 12 months*									0.055
No	791	18,869	167	3,843	20.4	624	15,026	79.6	
Yes	158	3,769	49	1,077	28.6	109	2,692	71.4	

N: expanded n from the sampling weights and design; %: proportion from N; χ ^2^ = p: Pearson’s chi-squared test; BMI: body mass index; CVD: cardiovascular diseases; CHF: congestive heart failure; MI: myocardial infarction; CVA: cerebrovascular accident

* The differences in relation to the total are due to the lack of information in the variable.

Statistically significant differences were observed in the distribution of CKD in the variables self-assessment of health, obesity according to the BMI, SAH, diabetes and dyslipidemia. Except for the self-assessment of health, all are components of metabolic syndrome, also related. The current use of medications also showed statistically significant difference in the presence of CKD (Table 3).

The assessment of odds ratio with the introduction of potentially confusing variables observed a statistically significant association between CKD and age group, self-assessment of health, obesity, hypertension, diabetes and metabolic syndrome, even after adjustment. The model for MS analysis did not include the variables SAH and DM, since they already belong to the definer set of the syndrome (Table 4).

**Table 4:** Factors associated to CKD in older adults from Rio Branco, state of Acre, 2014.

Variable	OR _crude_ (95%CI)	OR _adjusted_ (95%CI)
Age group (years)		
60–69	1	1
70–79	1.64 (1.12–2.40)	1.83 (1.25–2.68)
80 and more	3.20 (2.24–4.57)	3.99 (2.70–5.91)
Self-assessment of health		
Very good/Regular	1	1
Bad/Very bad	1.70 (1.12–1.58)	1.79 (1.10–2.91)
BMI (kg/m ^2^ )		
< 25.0	1	1
25.0–29.9	1.46 (0.95–2.24)	1.37 (0.90–2.08)
≥ 30.0	1.85 (1.11–3.08)	1.69 (1.02–2.80)
Use of medicines		
No	1	1
Yes	1.41 (1.03–1.94)	0.70 (0.43–1.14)
Arterial hypertension		
No	1	1
Yes	2.10 (1.22–3.63)	1.82 (1.04–3.19)
Diabetes		
No	1	1
Yes	3.39 (2.34–4.91)	3.39 (2.13–5.40)
Dyslipidemia		
No	1	1
Yes	1.59 (1.04–2.43)	1.43 (0.87–2.37)
Metabolic syndrome*		
No	1	1
Yes	2.27 (1.61–3.21)	2.49 (1.71–3.63)

BMI: body mass index; OR: odds ratio; OR _crude_: crude analysis; OR _adjusted_: adjusted analysis by the variables with each other

* In the analysis of metabolic syndrome, BMI, dyslipidemia, arterial hypertension and diabetes.

## DISCUSSION

The prevalence of CKD was high in older adults, being associated with age, self-assessment of health as bad or very bad, obesity, diabetes and metabolic syndrome, even after adjustments. In this research, the incorporation of objective criteria to standardize the staging of CKD (use of GFR according to the formula CKD-EPI and the classification of albuminuria) favored the comparison with different locations ^15^ .

The prevalence of CKD increases with advancing age ^16^ . The aging process results in reduced GFR, which is a normal biological phenomenon linked to cellular and organ senescence, resulting from the change in the volume of the kidney, with reduced number of nephrons, change in the vasoactive response and changes in the activity of the renin-angiotensin systems, associated to the cellular oxidative stress ^17^ . Another functional abnormality of aging is the increased permeability of the glomerular basement membrane, which allows the excretion of a larger number of proteins, including albumin, another factor that influences the increase of the prevalence of renal injury in older people ^18^ . The decline of renal function, which seems to start early in the second decade of life ^19^ , was observed in this study, with increased prevalence of CKD in each surveyed age group, reaching 37.8% in octogenarians.

The high prevalence of older adults with CKD, especially those with 70 years or more, according to the criteria of the Kidney Disease: Improving Global Outcomes (KDIGO) 2012, has been questioned by researches who defend a new definition of CKD for older adults, not classifying individuals in stage 3a as sick, but only those with GFR < 45 ml/min/1,73 m ^2^ and the presence of albuminuria ^20^ . This proposal would reduce the prevalence of renal injury in this study.

It is worth noting that two-thirds of the elderly population show reduced GFR without complications related to health in most cases; however, the presence of chronic conditions such as hypertension and diabetes, associated to aging, may lead to the sharp decline of renal function, with increased prevalence of CKD ^21^ . Different mechanisms are related with the physiopathology of CKD associated to SAH, including the deregulation of sodium content, renin-angiotensin system and the endothelial function ^22^ , which become more expressive with age.

In a national survey performed with 7,552 subjects in Italy, the prevalence of CKD in the age group from 60 to 69 years was 8.7%, and in the group from 70 to 79, it was approximately 17.0%, with hypertension associated to the disease (OR = 1.55) ^23^ . In a population study in Poland with 2,413 participants, the prevalence in the age group of 60 to 79 years was 15.3%, with a nearly twice as likely chance of the hypertensive individual being classified as having CKD ^24^ . In this study, hypertension was also positively associated with CKD.

In addition to hypertension, among the morbidities assessed in this research, diabetes obtained a greater association with CKD, which was also observed in other researches ^23^ ^,^ ^24^ . Among diabetics, the mechanisms involved in the renal lesion result in glomerular hyperfiltration with proteinuria — in most cases, glomerulosclerosis with reduced GFR ^25^ . In a study on mortality due to CKD in Rio Branco, diabetes and SAH were among the associated leading causes of death ^26^ .

In this study, bad and very bad self-assessment of health was also related to CKD. This assessment is useful as an overall measure of the health status in the general population, being consistent with the real state of health. In a population-based research performed in China, with individuals with 18 years or more, the presence of CKD resulted in a four times greater change of self-report to a poor health condition (OR = 4.41; 95%CI 3.20–6.07) ^27^ .

Obesity increases the risk of occurrence of diseases considered risk factors for the development of CKD, such as SAH and diabetes, in addition to acting in the progression of the stages of CKD by hyperfiltration, to meet the demands of the body weights and of the increase of the intra-glomerular pressure, which damages renal structures ^28^ . Among the older adults of this research, obesity defined by BMI, was associated with CKD.

The data analysis of 9,100 adults of the Chronic Renal Disease in Turkey (CREDIT) evidenced the association of CKD to obesity, determined by the BMI, and to metabolic syndrome (OR = 1.32; 95%CI 1.11–1.57) ^29^ . In this research, even after adjustment, the presence of CKD increased more than twice the chance the individual has of having metabolic syndrome.

One of the limitations of this research is the possible attenuation of the associations observed, due to the effect of survival among older adults. Another factor is the definition of CKD by the estimation of the GFR and albuminuria from a blood and urine punctual sample, since it is recognized that the confirmation of the disease occurs when the abnormality in GFR or albuminuria for a period of three months. In addition, the choice of more than one individual of a same household may result in the reduction of variability. It is necessary to emphasize, also, that the exclusion of individuals without physical or cognitive capacity to participate in the study may lead to errors in the estimation of the GFR, since these are more likely to reduce this rate.

Finally, the importance of population surveys stands out as an important source of information on the health of the older adult population, aimed at the definition of risk factors and the prevention of diseases or their complications. Finding the disease in its early stages among older adults must be a priority in Northern Brazil, which has an aging index that went from 8.2% in 1970 to 24.6% in 2010, in addition to the increase of chronic morbidities such as diabetes, SAH and obesity ^30^ , factors associated to CKD.
